# Toward Autonomous Extraction and Building of Machine-Readable Molecular Models from Publications Using Large Language Models

**DOI:** 10.1021/acs.jcim.6c02128

**Published:** 2026-10-06

**Authors:** Florian Fleckenstein, Volodymyr Rodin, Isabell Dieudonné, Fadi Al Machot, Silvia Chiacchiera, Martin Horsch, Amila Akagic, Simon Stephan

**Affiliations:** † Laboratory of Heat- and Mass Transfer, 9376 Otto von Guericke University Magdeburg, 39106 Magdeburg, Germany; ‡ Department of Computer Science and Informatics, 112354 University of Sarajevo, 71000 Sarajevo, Bosnia and Herzegovina; § Department of Data Science, 56625 Norwegian University of Life Sciences, 1432 Ås, Norway; ∥ 97005 UKRI Science and Technology Facilities Council, Daresbury Laboratory, WA4 4AD Warrington, U.K.

## Abstract

Force field models are one of the central pillars of molecular simulations. A large number of molecular force field models has been developed in the past decades—mostly published in scientific papers. Building machine-readable force field input files for simulation engines is a tedious and error-prone task. We developed a method for extracting and building force field files from publications using large language models (LLMs). We have tested the new method by extracting 114 force field models from 21 scientific publications. The studied force fields comprise 6–74 parameters that were automatically extracted, identified, annotated, and allocated. We have compared the performance of different LLMs, namely Gemini 2.5 Pro, Claude 4 Sonnet, Claude 3.7 Sonnet, Gemini 2.5 Flash, and GPT-4o. Overall, they yield similar performance—yet there are important differences in individual cases. The overall best performance was obtained by the Gemini 2.5 Pro LLM. The force field parameters were extracted and identified with an accuracy of 94.0% using the Gemini 2.5 Pro LLM. The new LLM-assisted extraction method drastically reduces the time required for building force field files. Nevertheless, human-in-the-loop verification is still indispensable.

## Graphical abstract

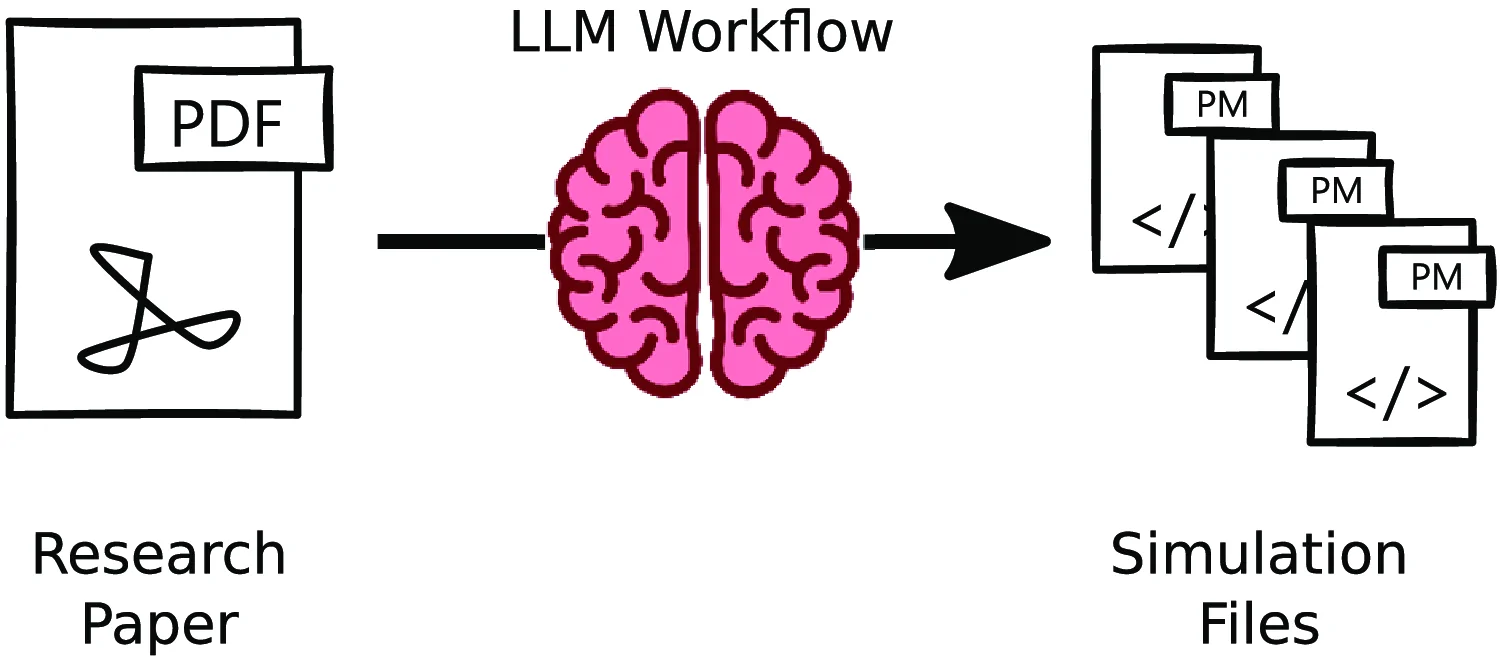

## Introduction

Simulations based on molecular models are a powerful tool that enables the prediction of macroscopic thermophysical properties as well as the modeling of nanoscopic processes. Classical molecular simulations, i.e., molecular dynamics (MD) and Monte Carlo (MC) simulations, have become a standard tool in many scientific disciplines such as computational physics,^1–3^ physical chemistry,^4–7^ tribology,^8^ molecular biology,^9–13^ astronomy,^14,15^ and chemical engineering.^16–21^

Matter is modeled in MD and MC simulations using molecular models—so-called force fields—that describe the interactions on the atomistic level. In MD simulations, the force fields are used to calculate the actual forces between interaction sites such that the trajectories of the interaction sites over a given time period can be computed. In MC simulations, the potential energy is used to evaluate the probability of existence of a given atomistic configuration by computing the total potential energy. A force field mathematically describes the molecular interactions—deployed either in MD or MC simulations. Molecular simulations can have impressive predictive capabilities, which facilitates their popularity. Nevertheless, the predictive capabilities depend on the quality of the employed force field to reflect reality regarding the molecular interactions.^22–29^ Moreover, it has been emphasized that the successful reproducibility of predictions from molecular simulations strongly depends on accurate force field models and that even minor mistakes by users can have severe consequences for the results.^30–32^ Reducing the influence of the simulator as an error source when building force field files and standardizing workflows is desirable.^32–35^

A large number of force fields have been proposed in the past decades, e.g., refs 36–39 . Moreover, various MD/MC simulation engines are in use today, e.g., refs 40–47 . For employing a given force field in a simulation engine, machine-readable force field input files are required. Force field models have been published mostly in classical journal articles in the past decades. In most cases, the force field specifications are reported in the main .pdf file of a given publication. Often, the information and data defining a given force field are spread across different entities of a paper, i.e., figures, tables, text, etc. Force field models are often described in text and tables, whereas ready-to-use simulation files are practically never provided in publications (partly due to the lack of standardized force field file formats). Moreover, a publication may report one or multiple force field models for one or more molecules and/or different molecules, with relevant details either consolidated (e.g., in a table) or scattered throughout the text, sometimes using different potential forms or unit systems. In some cases, relevant information might be included in a graphic such as geometry details. Building force field input files “by hand” by simulators is a tedious and error-prone task. While some force field databases exist, e.g., the MolMod database,^36,37^ that provide input files for simulation engines, these databases comprise only parts of the models available in the literature. Presently, force field models are not available under FAIR (Findable, Accessible, Interoperable, and Reusable) conditions.^48^ Digitizing, annotating, and depositing force fields in databases is still in its infancy. In this work, we propose a methodology for digitizing force field models from scientific publications using large language models (LLMs).

LLMs have gained significant attention across a wide range of natural language processing (NLP) tasks in both academia and industry.^49,50^ Their performance is being systematically evaluated in diverse applications to better assess their capabilities from multiple perspectives. It is evident that they will continue to play an increasingly important role at the societal level, as their potential and associated risks are more thoroughly understood. A key advantage of LLMs, compared with traditional approaches, lies in their versatility to address a broad set of tasks within a single framework. Fundamentally, LLMs are large-scale neural network models trained on extensive text corpora to learn patterns of language, semantics, and context.^51,52^ The core technology underlying most LLMs is the self-attention mechanism within the transformer architecture, which serves as a foundational component for language-related tasks.^53^ To date, LLMs have been successfully applied to tasks such as information extraction, semantic structuring, classification, summarization of unstructured data, and so on. These applications highlight their potential in the digitization and automated processing of documents and complex data.^54,55^

In this work, we propose a novel LLM-assisted methodology for digitizing force field models, as a step toward fully autonomous digitization. This new method is tested using five different LLMs and validated using a variety of force fields extracted from 21 publications from the literature. The results from the LLM-assisted digitization are compared with force field files from a benchmark database. This paper is organized as follows: First, a brief introduction to force field models is given—focusing on aspects relevant for their digitization from publications. Then, the methodology for digitizing force field models from publications is presented. Third, the results from the case study based on 21 publications comprising 114 (component-specific rigid classical) force field models are presented and discussed. Finally, conclusions are drawn.

## Structure of Molecular Force Field Models

There is a wide range of force field types available today. Force fields can be classified using different attributes, e.g., atomistic architecture (flexible or rigid), modeling approach (component-specific or transferable), model detail level (all-atom, united-atom, coarse grain), force field chemistry (reactive or nonreactive), underlying parametrization strategy (bottom-up or top-down), employed interaction potentials, etc. An in-depth discussion is, for example, given in ref 56. In this work, classical (i.e., nonreactive, pairwise-additive) component-specific rigid force fields for molecules are used for testing the new digitization method.

In classical force fields, the molecular interactions are modeled by interaction potentials that describe the potential energy as a function of the spatial relations between two (or more) particles/sites *u*( *r* ). Despite the fact that these interaction potentials provide a relatively crude simplification of the “true” molecular interactions, classical force fields have strong predictive capabilities.^57–60^ In classical molecular force field models, atoms (or groups of atoms) are represented as interaction sites, i.e., points in space. The interactions between these sites are described by the interaction potentials for which different ansatz functions exist, e.g., the Lennard-Jones (LJ) potential, multipole interactions, etc. In rigid force field models, the interaction sites of a model have fixed relative positions. In flexible force fields, intramolecular degrees of freedom exist, which require additional interaction potentials. In this work, rigid force field models were used for testing the proposed digitization method. An interaction potential (both intermolecular and intramolecular) in a force field is specified by the general mathematical equation and the specific parameters applicable to the interactions under consideration. Moreover, the mass of each site needs to be specified to solve the equations of motion in MD simulations, whereas it is not required in MC simulations, as atomistic configurations are sampled on the basis of the total potential energy. In summary, a force field consists of (a) the coordinates of the interaction sites and (b) the information on the interaction site type allocated to a given site, which is specified by the mathematical equation and the corresponding parameters. All this information has to be comprised in a well-defined way in force field files to be used by a simulation engine.

Intermolecular interactions are usually represented by dispersive-repulsive and electrostatic contributions. Dispersive-repulsive interactions between two particles *i* and *j* separated by a distance *r*_*ij*_ can, for example, be modeled by the Mie potential *u*_Mie_ (cf. eq 1) as

$$u_{\mathrm{Mie}}\left(r_{\mathrm{ij}}\right)=\frac{n}{n-m}{\left(\frac{n}{m}\right)}^{\frac{m}{n-m}}\varepsilon_{\mathrm{ij}}\left[{\left(\frac{\sigma_{\mathrm{ij}}}{r_{\mathrm{ij}}}\right)}^{n}-{\left(\frac{\sigma_{\mathrm{ij}}}{r_{\mathrm{ij}}}\right)}^{m}\right]$$ 1

where *n* and *m* denote the repulsive and dispersive exponents, respectively, ε_*ij*_ is the dispersion energy, and σ_*ij*_ is the size parameter of the interaction site. For the special case *n* = 12 and *m* = 6, the Mie potential reduces to the LJ potential.^61^

Electrostatic interactions are commonly described using either point charges, point dipoles, or point quadrupoles. For point charges, the electrostatic interaction is given by Coulomb’s law (cf. eq 2)^62^

2 $$u_{\mathrm{ij}}^{\mathrm{qq}}\left(r_{\mathrm{ij}},q_{i},q_{j}\right)=\frac{1}{4\pi \varepsilon_{0}}\frac{q_{i}q_{j}}{r_{\mathrm{ij}}}$$

where *q*_*i*_ and *q*_*j*_ describe the magnitude of the charge and *r*_*ij*_ is the distance between the two charges.

Point dipoles describe the electrostatic field induced by two point charges with equal magnitude, but opposite sign. The interaction of two point dipoles is given by eq 3 ^62,63^

3 $$u_{\mathrm{ij}}^{\mathrm{DD}}\left(r_{\mathrm{ij}},\theta_{i},\theta_{j},\phi_{\mathrm{ij}},\mu_{i},\mu_{j}\right)=\frac{1}{4\pi \varepsilon_{0}}\frac{\mu_{i}\mu_{j}}{r_{\mathrm{ij}}^{3}}\left(\sin\theta_{i}\sin\theta_{j}\cos\phi_{\mathrm{ij}}-2\cos\theta_{i}\cos\theta_{j}\right)$$

where μ_*i*_ and μ_*j*_ describe the magnitude of the point dipoles, *r*_*ij*_ the distance between the two point dipoles, and θ_*i*_, θ_*j*_, and ϕ_*ij*_ indicate the relative angular orientation of the two point dipoles, where θ_*i*_ and θ_*j*_ describe the angle of each dipole direction with regards to the distance vector between the two dipoles and ϕ_*ij*_ describes the azimuthal angle between the two dipoles.

Point quadrupoles describe the electrostatic field induced by either two collinear point dipoles with the same moment, but opposite orientation, or by at least three collinear point charges with alternating sign. The interaction potential of two quadrupole sites at a distance *r*_*ij*_ is given by eq 4 ^63,64^

4 $$\begin{array}{l} u_{\mathrm{ij}}^{\mathrm{QQ}}\left(r_{\mathrm{ij}},\theta_{i},\theta_{j},\phi_{\mathrm{ij}},Q_{i},Q_{j}\right)\\ =\frac{1}{4\pi \varepsilon_{0}}\frac{3}{4}\frac{Q_{i}Q_{j}}{r_{\mathrm{ij}}^{5}}\left[1-5\left(\cos^{2}\theta_{i}+\cos^{2}\theta_{j}\right)-15\cos^{2}\theta_{i}\cos^{2}\theta_{j}+2{\left(\sin\theta_{i}\sin\theta_{j}\cos\phi_{\mathrm{ij}}-4\cos\theta_{i}\cos\theta_{j}\right)}^{2}\right] \end{array}$$

where *Q*_*i*_ and *Q*_*j*_ are the magnitudes of the quadrupole moments, θ_*i*_, θ_*j*_, and ϕ_*ij*_ indicate the relative angular orientation of the two point quadrupoles,^63^ analogously to the description above for the point dipoles.

The number of parameters differs for the different interaction potentials, e.g., for a simple LJ potential, the two parameters ε_*ij*_ and σ_*ij*_ are required—each being a single scalar value. For a Mie potential, additionally *m* and *n* are required. For a point dipole, on the other hand, one scalar parameter value is required, namely the dipole moment μ, and one vector specifying its spatial orientation (θ and ϕ), given as polar and azimuthal angles with respect to a chosen reference frame (e.g., defined by two orthogonal Cartesian axes). An interaction site type is defined by its potential parameters together with the site coordinates (including possibly an orientation vector), which are typically reported in Cartesian form (*x*, *y*, *z*), and its mass.

These interaction site types are not exhaustive, but the vast majority of rigid force field models are built from these interaction potentials. The force field models used for testing the digitization method are built from the above-listed interaction potentials. Nevertheless, the LLM-assisted method developed in this work is not limited to these interaction sites.

Intramolecular interaction potentials define the molecular flexibility and allow for vibrations within molecules. These include bond, angle, torsion, and improper torsion terms. A model in which the molecular geometry is fixed and no vibrations occur is referred to as “rigid”. In the following, we restrict our approach to rigid models that incorporate both dispersive-repulsive and electrostatic interactions. Figure 1 illustrates two representative molecular force field models, namely for chlorine^65^ and acetone.^66^ A force field typically comprises the spatial coordinates of the interaction sites (*x*, *y*, *z*), the mass associated with each site (*m*), and interaction parameters required for the Mie/LJ and electrostatic potential functions (ε, σ, *m*, *n*, *q*, μ, *Q*). For implementation in molecular simulation engines, the force field description must usually conform to specific data formats, which may additionally require auxiliary information and metadata such as the number of sites assigned to each interaction type and which interaction type is applicable for a given model.

**Figure 1:**
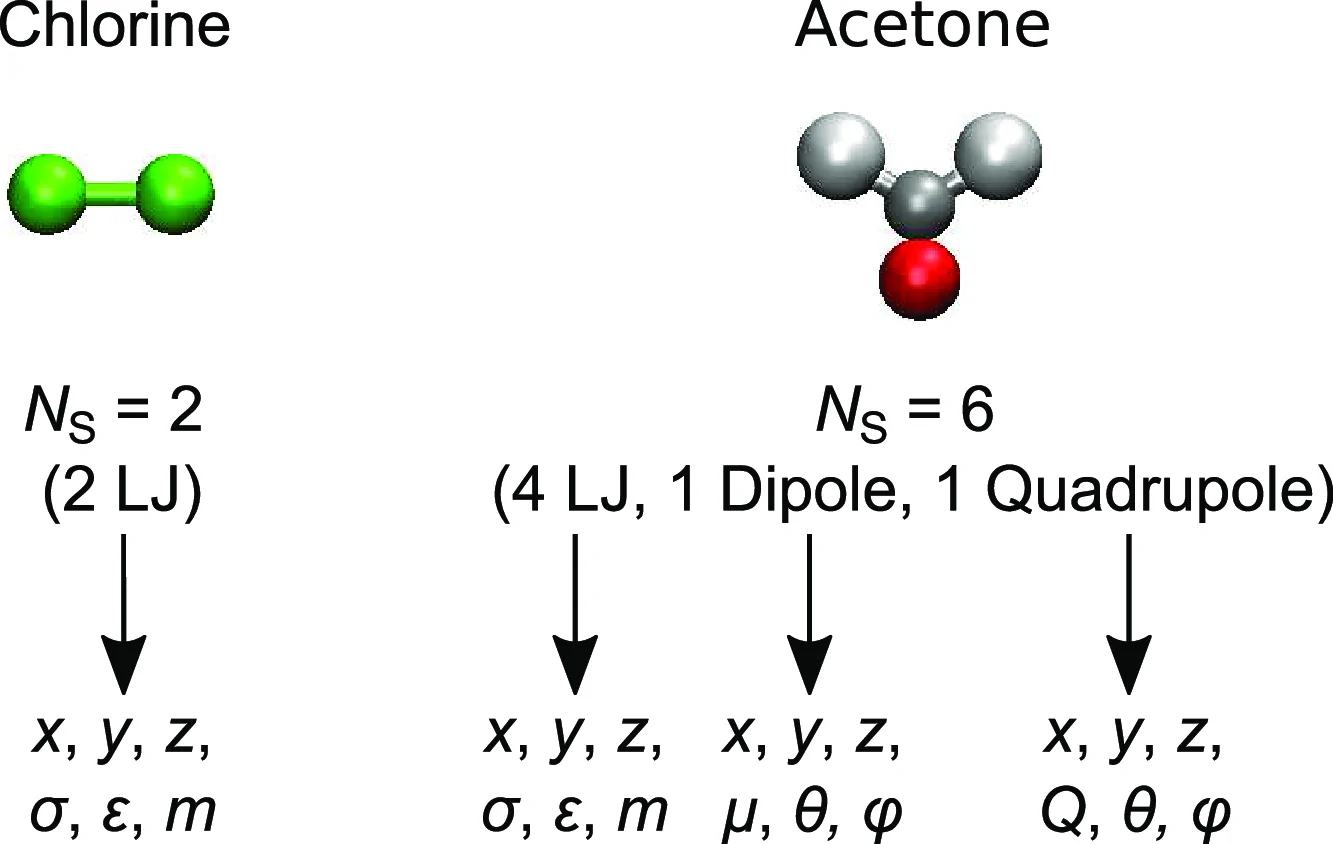
Schematic representation of two exemplary chosen molecular force field models with corresponding geometric, mass, and interaction parameters. Left: Chlorine model of *Romano& Singer*.^65^ Right: Acetone model of Windmann et al.^66^ The total number of parameters is 15 for chlorine (including 12 geometric, mass, and interaction parameters and 3 parameters required for the given file format used within the simulation engine) and 48 for acetone (including 36 geometric, mass, and interaction parameters and 12 parameters required for the given file format used within the simulation engine).

## Force Field Digitization Method

The LLM-assisted force field digitization method developed in this work extracts, transforms, identifies, and annotates information from scientific publications into machine-readable force field input files. We use the .pm file format as a target, which is already in use within simulation engines as well as data curation infrastructure^36,40^ —exemplary .pm files are given in the Supporting Information. The digitization method consists of a sequence of five steps: (1) Optical character recognition (OCR), (2) text cleaning, (3) table detection, (4) data extraction, and (5) file generation. Each step is described in detail, with an implementation provided in the Supporting Information. An overview of the entire pipeline is shown in Figure 2—in comparison to the classical manual approach. The goal of the workflow is to automatically extract and identify force field parameters from publications, standardize them into a machine-readable format, and produce reproducible force field files that include both the model and its metadata. We emphasize that Steps 1–5 execute without per-publication human intervention: OCR (Step 1) is used as provided, and the text-cleaning/table-detection rules (Steps 2–3) and extraction prompt (Step 4), though manually designed during development, require no further tuning for new publications. Human verification of the resulting parameter values is performed here against curated benchmark reference files to generate the accuracy metrics reported below, and, more generally, to help ensure the 100% accuracy required prior to any deployment in molecular simulation.

**Figure 2:**
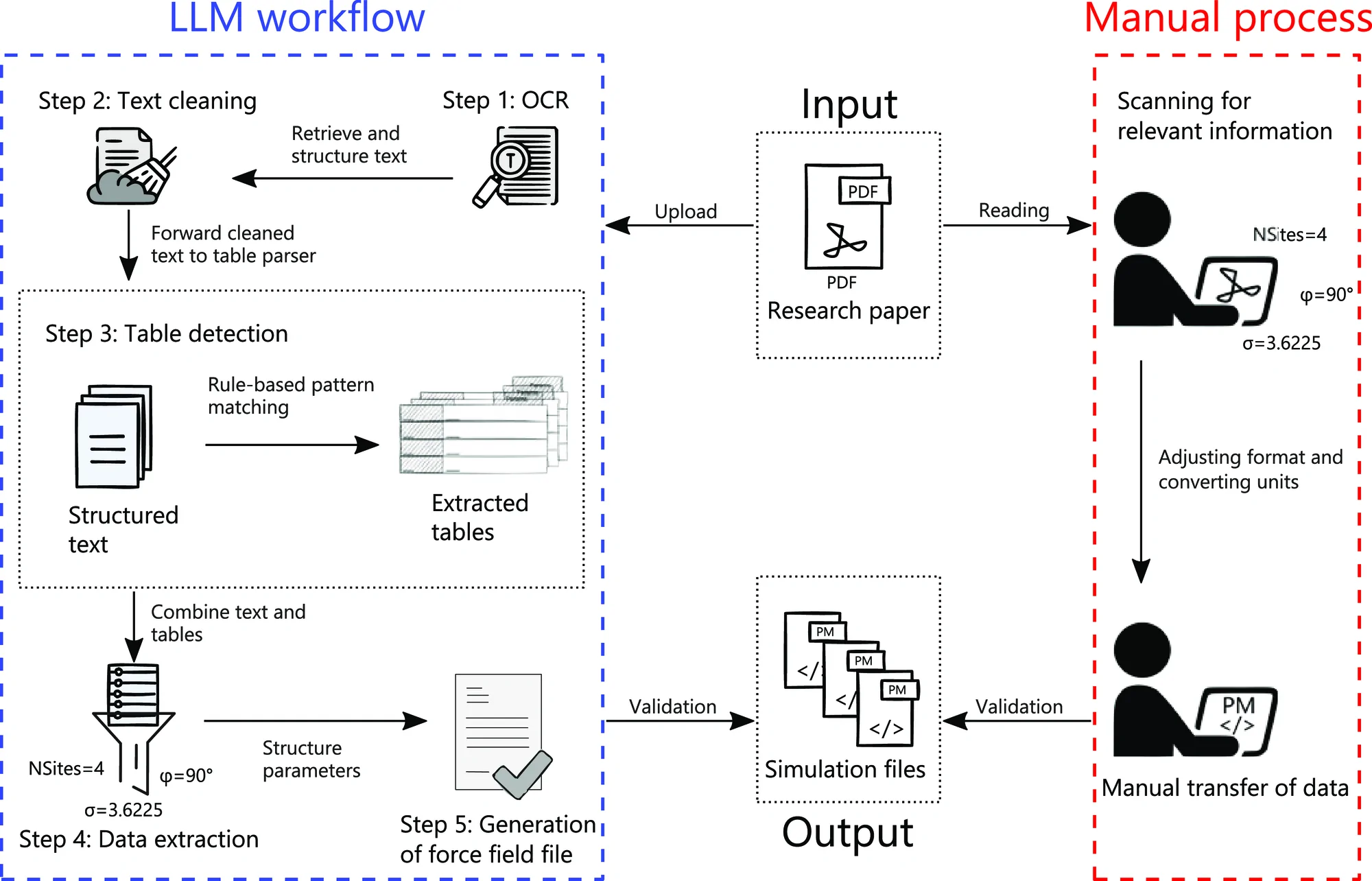
Overview of the automated data extraction pipeline (left) and the manual extraction process (right). Large Language Model (LLM) workflow: A research paper .pdf file is uploaded (Step 1) and OCR is used to retrieve and structure the text. The raw text is cleaned (Step 2) to remove nonessential artifacts, then rule-based pattern matching detects and extracts tables from the cleaned text (Step 3). Structured text and tables are merged for molecular parameter extraction (Step 4). These parameters are formatted and written as machine-readable .pm file (Step 5) and finally verified in a validation stage. Manual Process: Reading, scanning, and locating relevant information within the publication, mapping it to the simulation engine’s data scheme and format, and, if necessary, converting units. The data is then manually transferred to the .pm file and validated.

Manually creating an input file involves locating all relevant information within a given publication, mapping it to the simulation engine’s data scheme and format, and, if necessary, converting units (cf. Figure 2-right). Potentially, coordinates also have to be transformed to different coordinate systems, e.g., converting from Z-matrix to Cartesian coordinates. Evidently, units, notations, and conventions have to be interpreted correctly for extracting force field information. Ambiguities or missing details frequently require additional effort to resolve in the traditional manual route, and transcription into the precise syntax and unit system required by the simulation engine introduces further opportunities for error. Also, the validation of the constructed input file is not trivial since MD/MC simulations are in general costly and, moreover, simulation benchmark data are often scarce. As a result, preparing even a single ready-to-use input file can consume a considerable amount of time if done manually, e.g., some hours by an experienced simulator. LLM-assisted extraction offers an alternative by performing the same mapping and formatting tasks automatically and at scale, greatly reducing the required effort to reuse published force fields. In the LLM-assisted method developed in this work, all of these steps are carried out by the LLM pipeline.

### LLM-Assisted Force Field Extraction from Scientific Publications

The proposed approach automates the traditionally manual task of transcribing force field data from research papers into force field files. By reducing human involvement, the method circumvents user-induced transcription errors and accelerates data preparation. The workflow of the method is illustrated in Figure 2; it progresses through five steps, which are detailed in the following.

#### Step 1: Optical Character Recognition (OCR)

The process begins by selecting a research paper, typically in .pdf format, as the input. Many .pdf files, especially older scanned ones, store text as images rather than as selectable characters. To make the content usable by our method, the first step is to apply optical character recognition (OCR). For this purpose, we employ the *mistral-ocr-2505* model from Mistral AI,^67^ which acts as a digital reader by analyzing each page image, identifying the shapes of letters and numbers, and converting them into machine-readable text. Conceptually, this step transforms the visual representation of the document into structured textual data that is processed in subsequent steps of the pipeline process.

#### Step 2: Text Cleaning

The raw text obtained from OCR often contains nonessential elements. Therefore, text cleaning is applied. In this step, the system processes the raw digital text (as shown in Figure 3a) to remove artifacts that are not part of the main textual content or tables relevant for parameter extraction. This includes filtering out footers, bibliographies, metadata, and other content that do not contain relevant data. The purpose of cleaning is to ensure that only the core scientific text and data tables are passed on to the next step, preventing interference with subsequent analysis steps. An example of extracted text after text cleaning is illustrated in Figure 3b. In this example, the remaining text includes all sections of the paper from the introduction to the conclusion.

**Figure 3:**
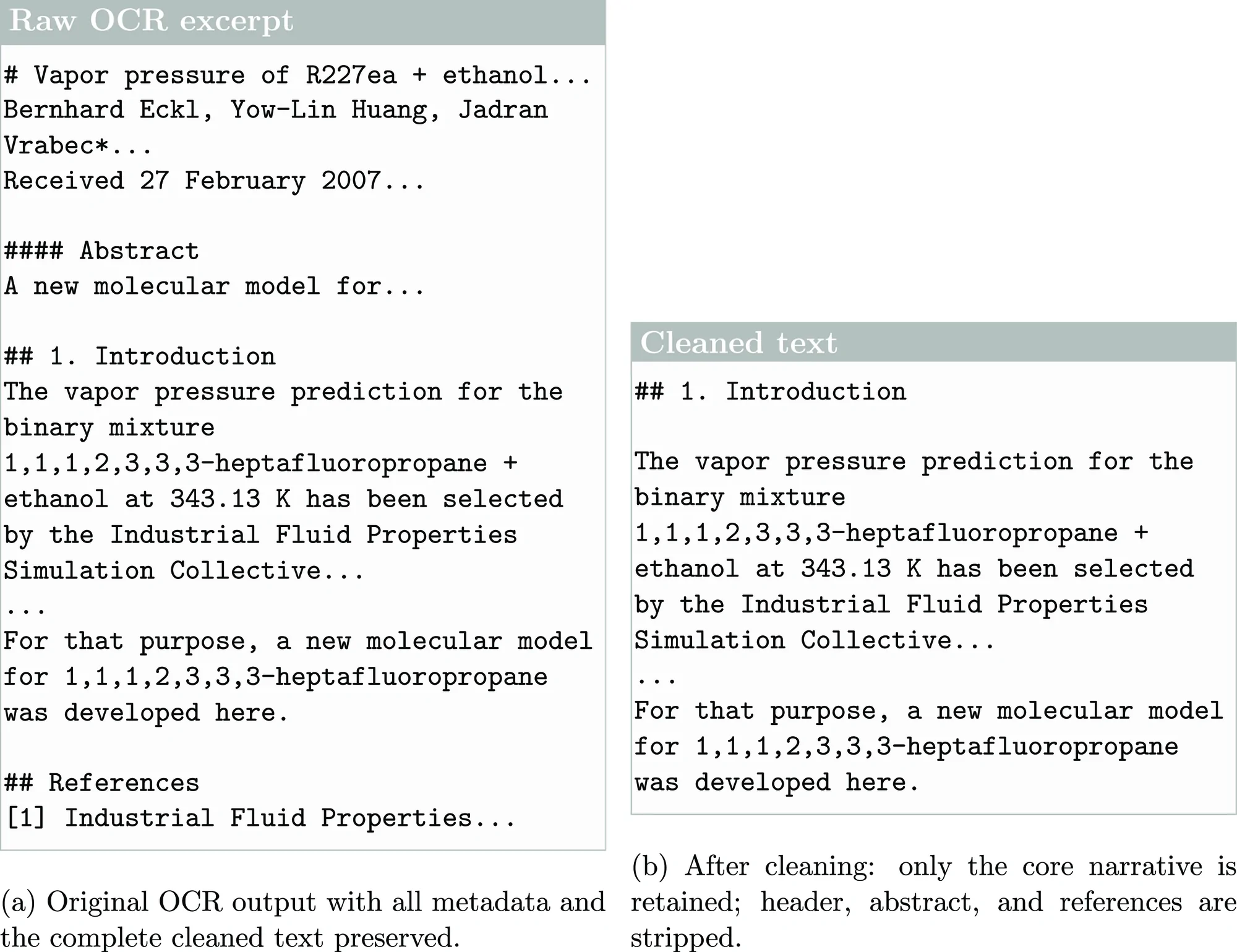
Text cleaning example: left shows the original OCR output including metadata, while right shows only the stripped version.

#### Step 3: Table Detection

In scientific publications, content of a force field can be presented in tables, figures, or in plain text. Moreover, multiple force fields can be specified in a single paper. The most frequent representation of force field information is tables, which we focus on in the developed extraction method. Step three focuses on table detection. Using rule-based pattern matching, the system scans the cleaned text specifically looking for structures that resemble tables. This involves searching for textual cues, such as captions explicitly labeling a table (e.g., “Table 1”, “Table II”), and recognizing the characteristic grid-like layout often formed by columns separated by spaces or vertical lines (i.e., |). By identifying these patterns, the method can distinguish tables from narrative paragraphs and extract their content while preserving the essential row and column structure. This results in two sets of data: The main body text and the structured content of the detected tables.

#### Step 4: Data Extraction

Having the cleaned text and identified tables accessible, the actual data extraction is carried out in Step 4. Here, LLMs^50^ are employed. The data extraction process is illustrated in Figure 4. Along with the prompt, the cleaned text and extracted tables are provided to the LLM as context. The LLM reads this context and uses its understanding of language and instructions to locate the relevant values and populate the required .pm file structure, associating the correct values with their corresponding parameters (e.g., linking a numerical value from a table column to the correct parameter name like “sigma” or “dipole” for a specific chemical site). This process resembles instructing an expert assistant to fill out a detailed form using information found within a document.

**Figure 4:**
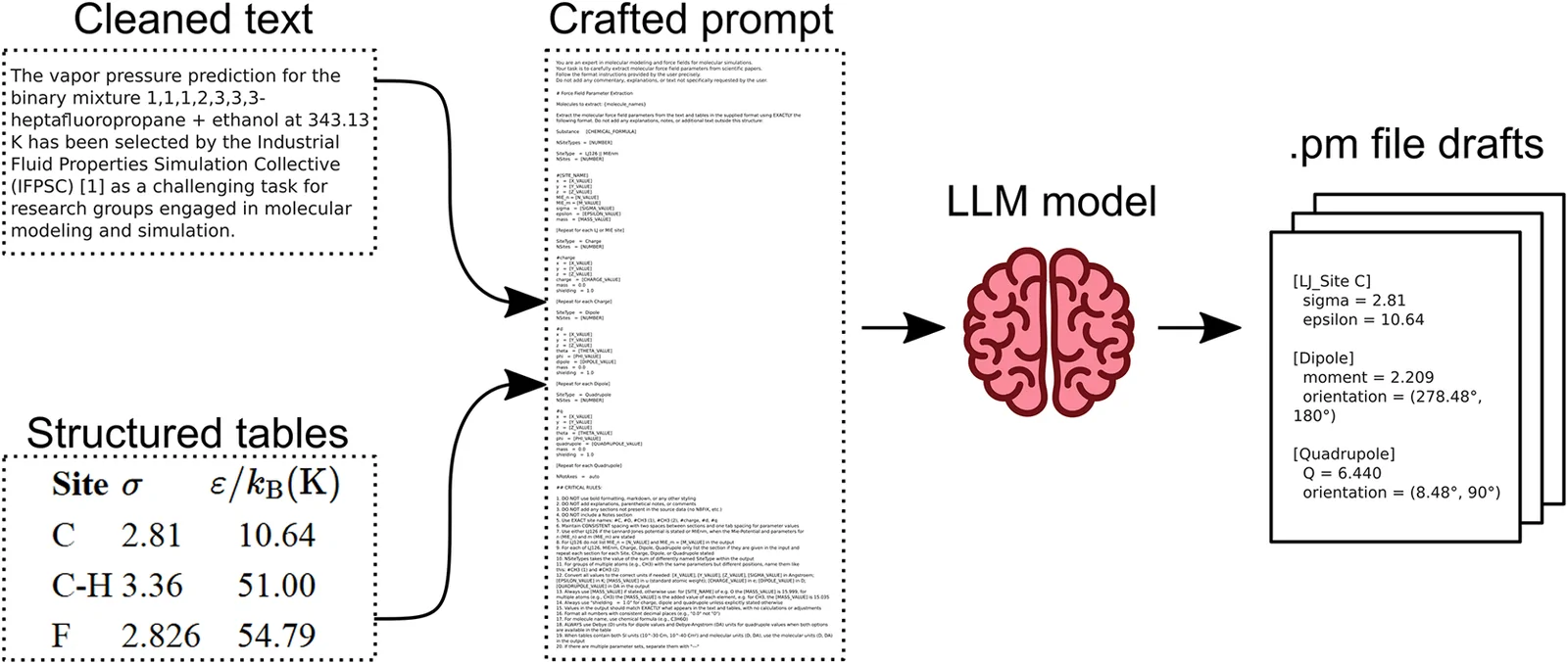
Schematic representation of the workflow: Cleaned text and the structured tables are fed, along with an expertly crafted prompt, into an LLM which acts as a molecular modeling expert and outputs a .pm file with extracted parameters (Lennard-Jones site, dipole, and quadrupole with orientations etc.).

Several LLMs were tested and compared as described below. Instead of programming explicit rules for every possible parameter, we provide the LLM with carefully crafted prompt instructions. This prompt guides the LLM, telling it to act as an expert in molecular modeling and instructing it on exactly what information to find (e.g., chemical names, specific parameters like σ, ε, dipole moment, etc.) and how to format this information according to the syntax of the .pm file format. Additional simulation running and sampling parameters (e.g., “shielding” in the simulation engine *ms*2) that cannot be inferred from the publication are set to default values to ensure completeness of the employed file format, but are explicitly excluded from evaluation (cf. Supporting Information).

The .pm file format uses Cartesian coordinates. In many force field publications, Cartesian coordinates of interaction sites are not provided directly; instead, structural information is often given in terms of distances and angles between sites (e.g., the Z-matrix form). Since the .pm format requires Cartesian coordinates, the LLM is tasked with producing *x*, *y*, *z* positions even when only Z-matrix data are available, without receiving explicit instructions on how to perform this translation. The correct unit system is also specified in the prompt (e.g., dipole moments μ must be expressed in Debye). Importantly, no additional metadata such as the total number of force fields in a publication or the number of interaction sites is inferred beyond what is explicitly reported.

#### Step 5: Writing Force Field File

The final step of the pipeline is the generation of machine-readable .pm files. After the extraction step, the parameters are organized into a standardized format intended for use in molecular simulation software or a force field database. During this process, the method retrieves the title of the source publication together with the names of the identified substances, which are included in the file header to preserve provenance and traceability. For each substance, a separate .pm file is created that comprises the generated header with the cleaned and structured parameter contents of the force field. The parameters are annotated according to the interaction site type. Thus, the resulting files encode not only the molecular model itself, but also the associated metadata.

## Validation

### Validation Procedure and Test Set

To assess the accuracy and reliability of the LLM-assisted digitization method, we tested multiple LLMs and papers for extracting force field data and creating .pm files. The content of the automatically created force field files was systematically compared against manually curated ground-truth reference files taken from ref 36 . On the basis of a comprehensive evaluation of the results, individual shortcomings of the new method were identified, which also provide insights into the inner workings of the LLM approach.

We employed five state-of-the-art LLMs: Gemini 2.5 Pro,^68^ Claude 4 Sonnet,^69^ Claude 3.7 Sonnet,^70^ Gemini 2.5 Flash,^68^ and GPT-4o.^71^ A brief description of the characteristics of these models is provided in Table 1. With the exception of the GPT-4o model, which was introduced in 2024, all of the other models were released in 2025. The selected LLMs share several common characteristics. They are all proprietary models with undisclosed parameter counts, reflecting a trend in commercial LLM development to focus on capability disclosures rather than architectural transparency. The models differ in their modality support, context window size, and output capacity. Gemini 2.5 Pro and Gemini 2.5 Flash offer the largest publicly stated context limits, clearly surpassing the limits of the other employed models. The maximum output length also varies, with Gemini 2.5 Pro/Flash and the Claude models supporting similar token numbers, whereas GPT-4o supports significantly smaller values (cf. Table 1). These differences suggest that the five models are optimized for different operational tasks, and their results may therefore vary significantly for specific tasks.

**Table 1:** Technical Specifications of Employed LLMs

**model**	**modality**	**max context/tokens**	**max output/tokens**
Gemini 2.5 Pro^68^	text, vision, audio, video	1,048,576	65,535
Claude 4 Sonnet^69^	text, vision	200,000	64,000
Claude 3.7 Sonnet^70^	text, vision	200,000	64,000
Gemini 2.5 Flash^68^	text, vision, audio, video	1,048,576	65,535
GPT-4o^71^	text, vision, audio	128,000	16,384

For testing and validating the developed method, we selected a set of 21 scientific publications comprising a total of 114 force field models. The publications were selected based on the force field compilation in the MolMod database^36,37^ covering force fields from more than 200 publications. Only publications with rigid component-specific force fields were considered here. Table 2 gives an overview of the *N*_pub_ = 21 publications used in the test (the numbering column “No” is used throughout this work for referring to specific papers). The set of 21 publications is a diverse compilation covering a wide range of years of publication, number of force fields in a given paper, research groups developing the models, molecule size etc. The number of force field models *N*_M_ per paper ranges from 1 to 62. Figure 5 shows histograms of properties of the studied force fields, i.e., number of interaction sites per force field *N*_S_ and number of parameters per force field *N*_P_. For the actual evaluation of the digitization method, the .pm files stored in the MolMod database were used as the ground-truth.

**Figure 5:**
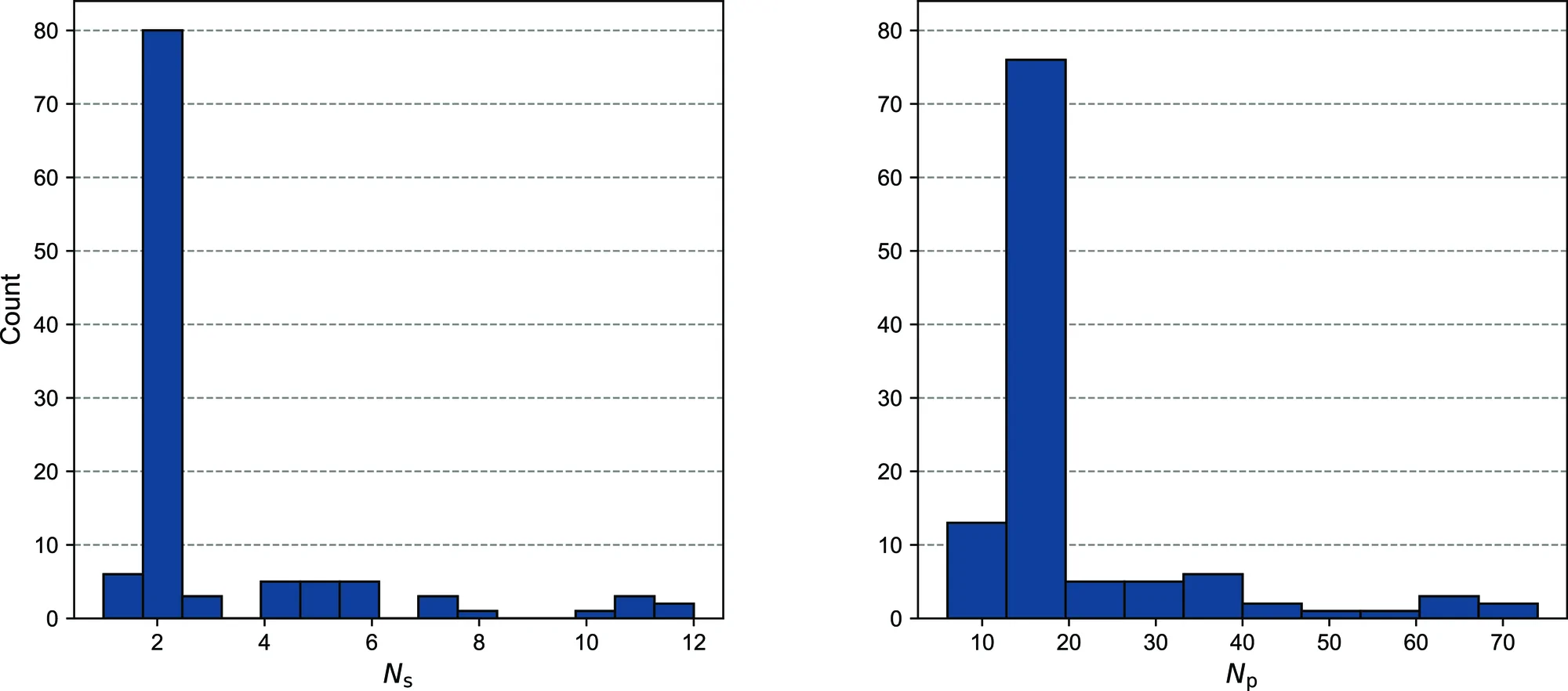
Histograms of number of interaction sites *N*_S_ (left) and force field parameters *N*_P_ (right) of the 114 force field models in the validation data set.

**Table 2:** List of Publications Used in the Validation Data Set, Ordered by the Sequence Used in the Analysis Charts^a^

**no.**	**paper**	**year**	*N*_**M**_	**no.**	**paper**	**year**	*N*_**M**_
1	Abbas & Nordholm^72^	1995	3	12	Merker et al.^73^	2012	2
2	Eckelsbach et al.^74^	2014	1	13	Miroshnichenko et al.^75^	2013	2
3	Eckl et al.^76^	2007	1	14	Muñoz-Muñoz et al.^77^	2017	3
4	Eckl et al.^78^	2008	10	15	Muñoz-Muñoz et al.^79^	2018	1
5	Fischer et al.^80^	1984	7	16	Romano & Singer^65^	1979	2
6	Gao et al.^81^	1997	8	17	Vrabec & Fischer^82^	1995	1
7	Horsch et al.^83^	2010	1	18	Windmann et al.^66^	2013	1
8	Kohns et al.^84^	2017	2	19	Zhang & Siepmann^85^	2006	1
9	Koyama et al.^86^	2005	1	20	de Santis et al.^87^	1986	3
10	Köster et al.^88^	2018	1	21	van Leeuwen^89^	1994	62
11	Merker et al.^90^	2010	1				

a The columns indicate the publication identifier number, year, and number of force field models (*N*_M_).

Algorithm 1 (Figure 6) outlines the evaluation workflow. The algorithm iterates over each ground-truth parameter (each associated with its corresponding interaction site), checking whether the model output contains the correct value, an incorrect value, or no value at all. On the basis of these checks, the proportion of correct matches is computed, returning both the extraction accuracy percentage and detailed counts for correct, incorrect, and missing parameters. Thus, we calculated three categories of values: correct, incorrect, and missing. Incorrect values contain some type of recognition error, while missing values were not identified at all. In the following, any deviation from the corresponding ground-truth value is considered incorrect, as no numerical tolerance is applied, except for the mass parameter, which is considered correct if it matches the atomic mass to within two decimals, to account for values inferred from molecular structure as the mass is often not explicitly stated in the corresponding publications.

**Figure 6:**
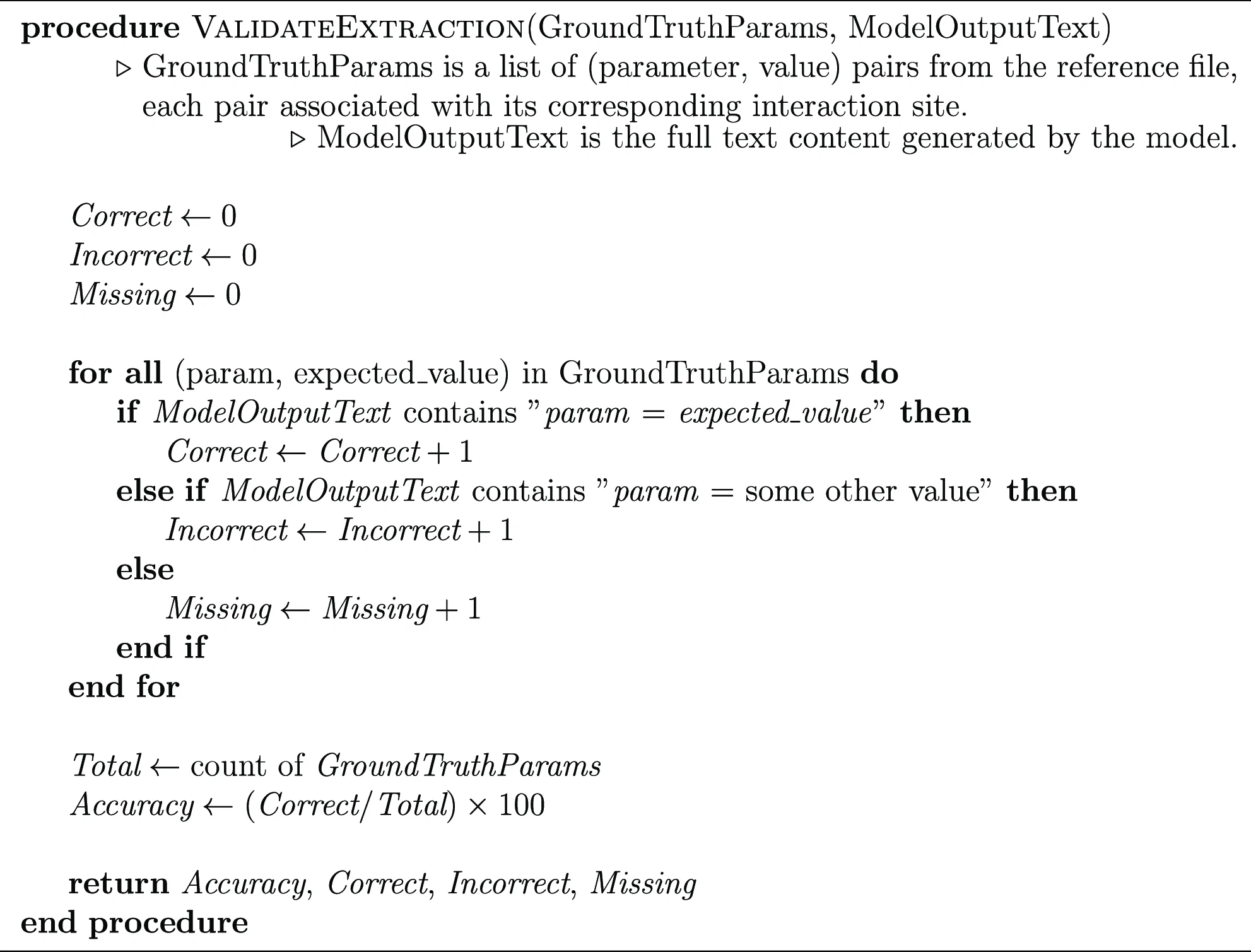
Algorithm of the core parameter validation logic

For the evaluation, the following metrics were used: per-paper extraction accuracy (*Acc*), average extraction accuracy (*Avg Acc*), precision (*P*), recall (*R*), and *F*1-score.^91^ We also report aggregated totals of correct (*N*_correct_), incorrect (*N*_incorrect_), and missing (*N*_missing_) values across all publications *N*_pub_ and runs *N*_run_ for each LLM. The digitization method was run *N*_run_ = 3 times for each studied LLM. These runs were independent, i.e., single end-to-end executions for a given paper and model using identical inputs, prompts, and hyperparameters; runs were isolated and differed only due to model stochasticity (e.g., sampling seed).

The per-paper parameter extraction accuracy by model and run (cf. eq 5) is calculated as

5 $$\mathrm{Acc}=\frac{1}{N_{\mathrm{run}}}\sum_{i=1}^{N_{\mathrm{run}}}\frac{N_{i,\mathrm{correct}}}{N_{i,\mathrm{correct}}+N_{i,\mathrm{incorrect}}+N_{i,\mathrm{missing}}}$$

where the index *i* denotes the totals for correct, incorrect, and missing values for a given publication and run *i*—contrary to the calculation of precision, recall, and *F*1-score, which are computed from aggregated totals across all publications.

The average extraction accuracy for a given LLM is calculated as the mean extraction accuracy over all publications (cf. eq 6) as

6 $$\mathrm{Avg}\;\mathrm{Acc}=\frac{1}{N_{\mathrm{pub}}}\sum_{i=1}^{N_{\mathrm{pub}}}\mathrm{Acc}$$

The precision (*P*) is the fraction of retrieved parameters that are correct and is computed from the aggregate totals (cf. eq 7) as

7 $$P=\frac{N_{\mathrm{correct}}}{N_{\mathrm{correct}}+N_{\mathrm{incorrect}}}$$

The recall (*R*) is quantified as the fraction of correct parameters that were successfully retrieved in relation to the total number of parameters that should have been identified, aggregated over all publications and runs (cf. eq 8) as

8 $$R=\frac{N_{\mathrm{correct}}}{N_{\mathrm{correct}}+N_{\mathrm{incorrect}}+N_{\mathrm{missing}}}$$

The *F*1-score is an aggregated measure of the precision and recall and is defined as the harmonic mean of these (cf. eq 9)­

9 $$F1=2\frac{P\cdot R}{P+R}$$

High values of precision and recall (close to 1) indicate strong performance. Deviations from unity highlight different types of errors: Precision decreases when parameters are retrieved but incorrect, whereas recall decreases when parameters are either extracted with incorrect values or not retrieved at all. The *F*1-score balances these two measures, which can be particularly useful when precision and recall do not correlate or inversely correlate.

## Results

Table 3 summarizes the performance of the different LLMs in terms of average extraction accuracy, precision, recall, *F*1-score, and average time per extracted model $\overset{®}{t_{M}\;}$ . Gemini 2.5 Pro achieved the highest average extraction accuracy at 94.0%. Claude 4 Sonnet and Claude 3.7 Sonnet followed with average accuracies of 92.3 and 91.9%, respectively. Gemini 2.5 Flash (89.9%) demonstrated competitive performance, while GPT-4o yielded the lowest results among the tested models (76.6%).

**Table 3:** Summary of Performance Metrics Results for Studied LLMs from the Validation Data Set^a^

**model**	**Avg Acc**/%	***P***/%	***R***/%	***F*1**/%	**correct**	**incorrect**	**missing**	$\overset{®}{t_{M}\;}$ /**s**
Gemini 2.5 Pro	**94.0**	**97.1**	**96.2**	**96.7**	7859 (96%)	231 (3%)	76 (1%)	15
Claude 4 Sonnet	92.3	96.2	96.0	96.1	7838 (96%)	310 (4%)	18 (0%)	9
Claude 3.7 Sonnet	91.9	94.8	94.5	94.7	7719 (95%)	420 (5%)	27 (0%)	9
Gemini 2.5 Flash	89.9	95.5	87.8	91.5	7168 (88%)	336 (4%)	662 (8%)	5
GPT-4o	76.6	93.3	68.7	79.2	5614 (69%)	401 (5%)	2151 (26%)	5
Average					(89%)	(4%)	(7%)	

a The average accuracy was computed by calculating the mean accuracy over papers; correct/incorrect/missing counts are aggregate totals of parameters across all papers and runs. The average time per extracted model $\overset{®}{t_{M}\;}$ was computed from the total extraction time of a run, aggregated over all 21 papers, divided by the number of extracted models

Given that one publication (#21, cf. Table 2) accounts for 62 of the 114 tested force field models and approximately 40% of the total parameters, we assessed the sensitivity of the reported results to this publication’s influence by reporting the metrics given in Table 3 excluding publication #21 (cf. Supporting Information). The overall extraction accuracy remains close to the values reported above. However, small differences in the performance of the different LLMs are noticeable. The three best-performing models (Gemini 2.5 Pro, Claude 4 Sonnet, Claude 3.7 Sonnet) show slightly lower accuracy, while the two lowest-performing models (Gemini 2.5 Flash, GPT-4o) show slightly higher accuracy, when publication #21 is excluded—mostly related to issues detecting dipoles and the corresponding parameters in these force fields. Despite these shifts, the relative ranking of all five LLMs remains unchanged.

The analysis of correct, incorrect, and missing values shows that Gemini 2.5 Pro, Claude 4 Sonnet, and Claude 3.7 Sonnet identified almost all parameters (i.e., less than 1% missing values for Gemini 2.5 Pro, Claude 4 Sonnet, and Claude 3.7 Sonnet). In contrast, the shares of missing parameters were 8 and 26% for Gemini 2.5 Flash and GPT-4o, respectively. This is reflected in the high (and similar) precision, recall, and *F*1-scores for the three best-performing models.

GPT-4o yields a high share of incorrect values (5%) and a substantially higher share of missing values (26%), resulting in the lowest precision, recall, and *F*1-score, which suggests greater difficulty in parameter interpretation and retrieval. Gemini 2.5 Flash followed with 4% incorrect and 8% missing values. This is in line with results reported by *Polak and Morgan*,^54^ who studied the extraction of material data from publications and obtained precision and recall values also around 90%. In their work, the LLMs retrieved material properties as structured triplets, and the good performance was achieved in part by asking follow-up questions and explicitly retrieving information on the uncertainty of the extracted data.

The execution time of the LLM-assisted method is similar for the different LLMs—around 5–15 s per force field (Gemini 2.5 Flash and GPT-4o being the fastest, and Gemini 2.5 Pro being the slowest). This time is evidently significantly shorter than the time a human would need to extract the information, apply possible conversions, and create a .pm file. While it is impressive that .pm files can be, in general constructed from .pdf files using LLMs, the average extraction accuracy of 94% (for the best-performing model, Gemini 2.5 Pro) approaches the reliability needed for direct employment in molecular simulation—with Gemini 2.5 Pro yielding force field files in which every parameter was correctly extracted in 71% of the tested cases—though human verification remains advisible to ensure 100% accuracy. For better understanding the role required by the human in the loop, a detailed analysis of the results was carried out to identify and understand typical errors of the LLM-assisted extraction method (see below).

Figure 7 shows the extraction accuracy per paper averaged over the three replica runs for each model. The overall mean extraction accuracy, and the range of scattering of the different LLMs, is depicted in Figure 8. Gemini 2.5 Pro regularly achieves the highest or nearly highest score in most papers, achieving 100% accuracy in several cases. Claude 4 Sonnet and Claude 3.7 Sonnet follow as the next best performers, delivering consistently good results with only a marginal difference between them. Gemini 2.5 Flash performs well on average but shows higher variability, while GPT-4o consistently underperforms the other models across many of the evaluated papers (e.g., scoring only 34% on paper #5 and 24% on paper #6). Possibly, the superior accuracy of Gemini 2.5 Pro stems from its design as a high-reasoning model, which allocates more computation during inference to produce robust and well-reasoned responses.^68^ In contrast, models optimized purely for speed may be less suited for tasks that demand strong generalization and domain knowledge, although Gemini 2.5 Flash performed surprisingly well, given its efficiency focus.

**Figure 7:**
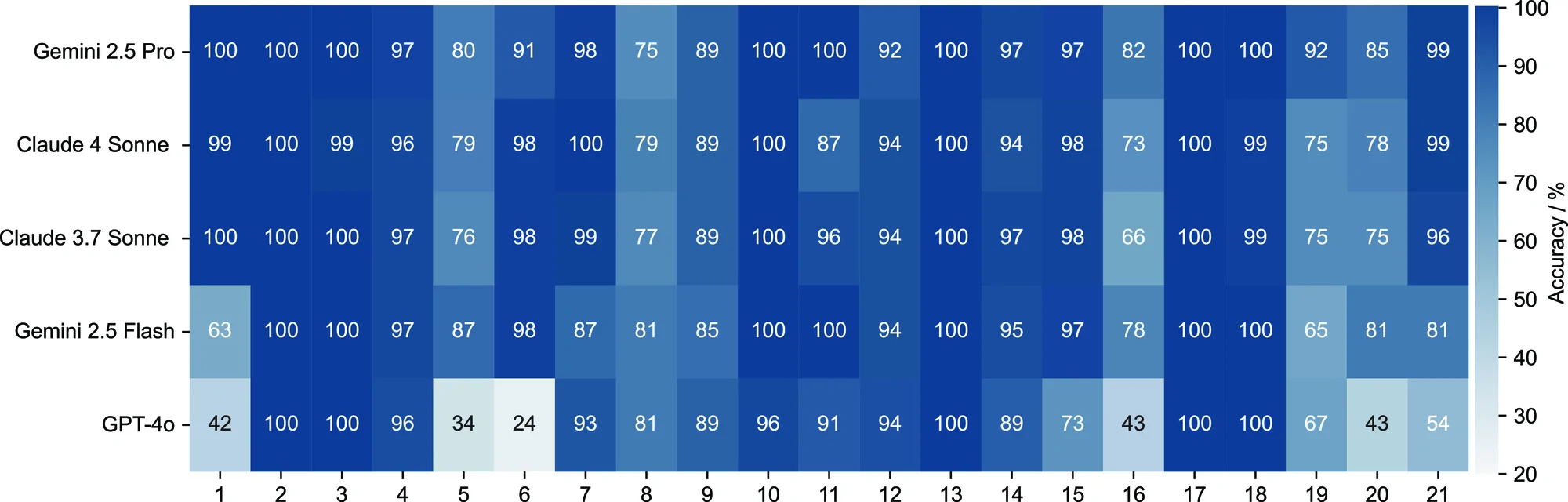
Per-paper extraction accuracy (cf. eq 5) averaged over three replica runs for each LLM. The color code indicates the accuracy.

**Figure 8:**
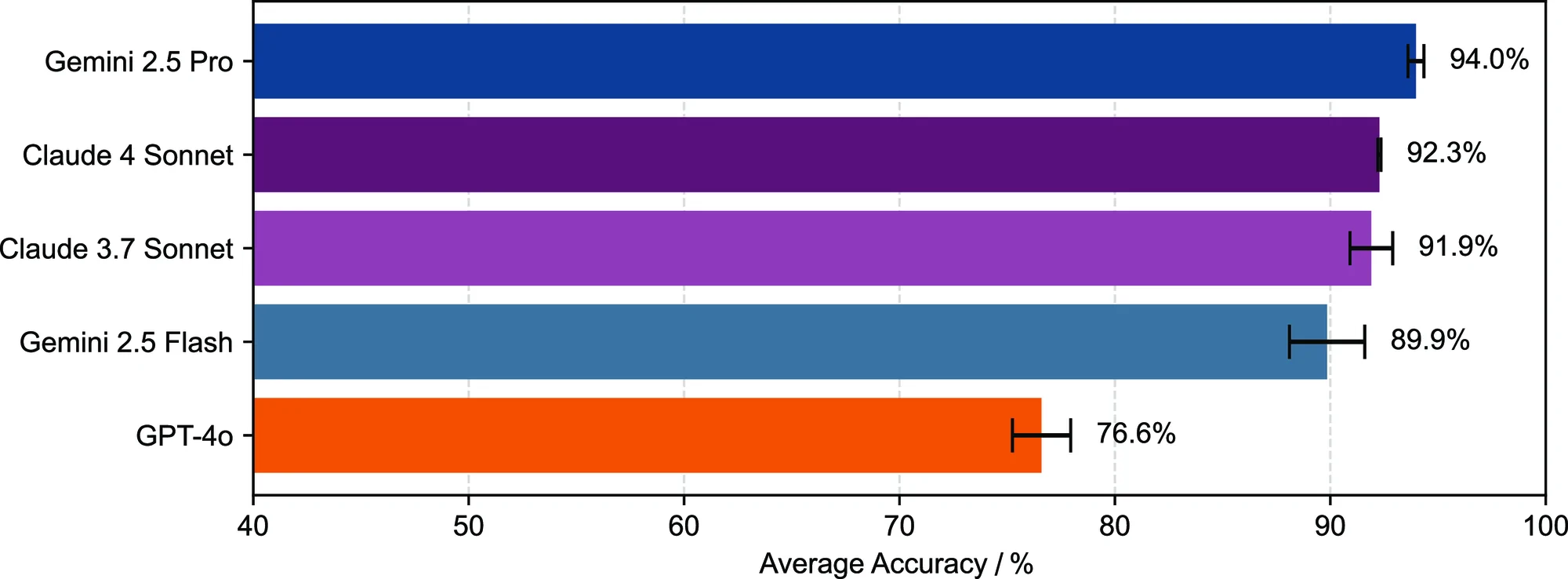
LLM extraction accuracy with run-to-run variability. Error bars indicate the variation across three independent replica runs, representing the mean performance between the best- and worst-performing runs for each paper.

For all employed LLMs, the obtained extraction accuracy differs for different papers, cf. Figures 7 and 8. This scattering is particularly notable for GPT-4o and Gemini 2.5 Flash. Moreover, the performance of the LLMs for a given paper differs significantly in part. For instance, Gemini 2.5 Pro, Claude 4 Sonnet, and Claude 3.7 Sonnet yield excellent results for paper #1 (99–100%), for which Gemini 2.5 Flash and GPT-4o yield relatively poor results (63 and 42%, respectively). Conversely, for paper #8, all models struggled to varying degrees, with accuracies around 75–81%.

Figure 9 shows per-paper, per-run extraction accuracy results for each model, with the global best across models shown in the background. In some cases, the results from the three independent runs are practically identical, e.g., for the #1 results from Gemini 2.5 Pro. In other cases, significantly different results are obtained from the three independent runs, e.g., for the #20 results from GPT-4o, where one run dropped significantly compared to the others. Gemini 2.5 Pro frequently meets or approaches the global best, reinforcing its leading position. Claude 4 Sonnet and Claude 3.7 Sonnet followed closely. The remaining models have for specific papers difficulties, where the extraction accuracy is significantly lower or more unstable across runs.

**Figure 9:**
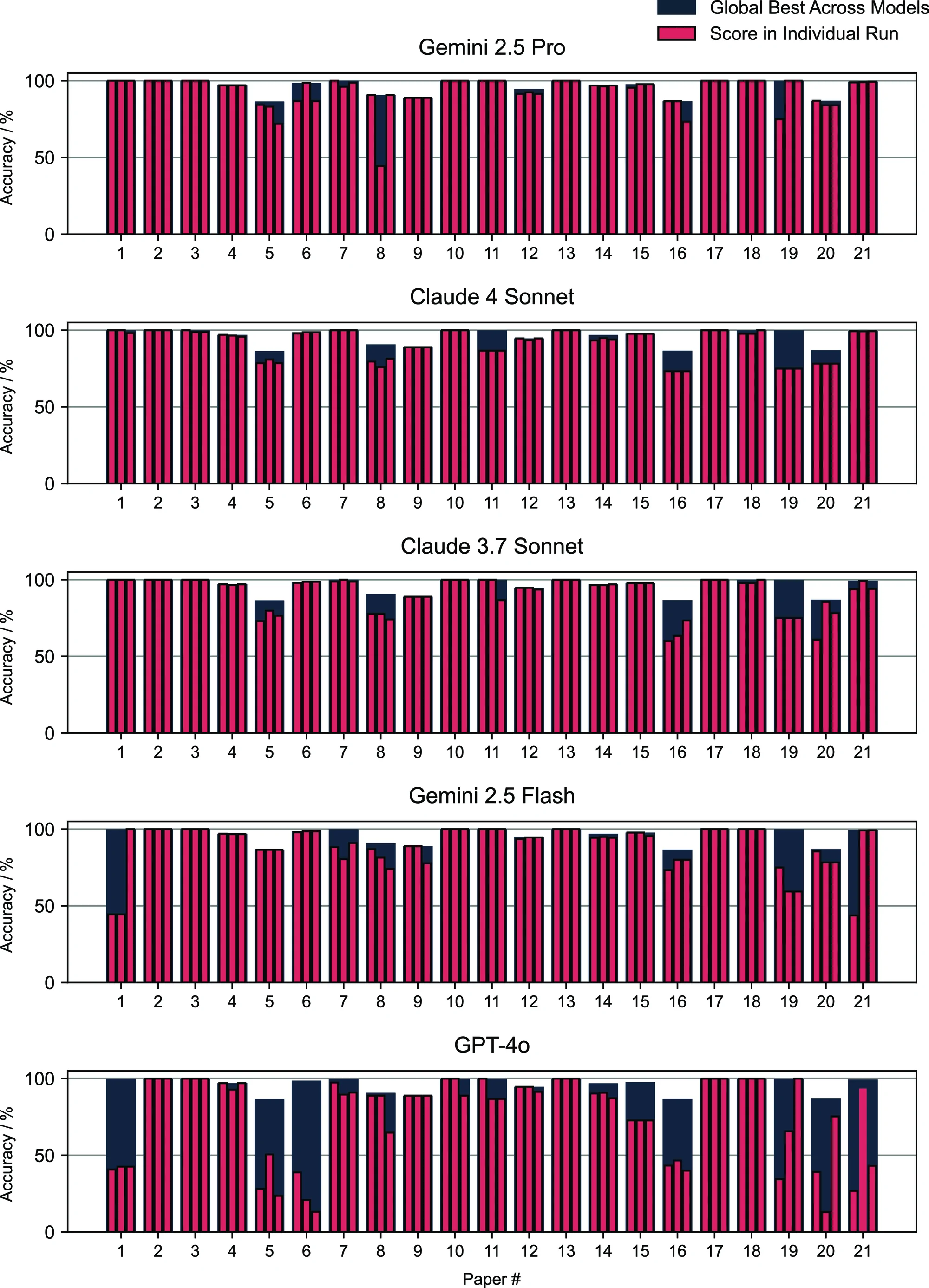
Per-paper parameter extraction accuracy by model and replica run (cf. eq 5), with the global best across models indicated in the blue background. Each group of three red bars for a paper represents the results from three individual runs of a given LLM.

To establish a better understanding of the nature of the extraction errors, we performed a detailed analysis of 4,632 errors across all employed LLM models, replica runs, force fields, and papers, using the error types defined in Table 4. The distribution, shown in Table 5, reveals that the two most dominant error types are “Missing Parameter” (63.3%) and “Numeric Value Mismatch” (10.1% of all errors). Other significant error types include “Count/Index Error” (9.3%) and “Coordinate Extraction Error” (9.0%).

**Table 4:** Classification of Extraction Errors Observed in Incorrect Results across Evaluated Datasets^a^

**error type**	**description**	**example (reference** → **extracted)**
numeric value mismatch	extracted numeric value differs significantly from reference.	mass = 127.91241 → 36.46 (Abbas, 1995)^72^
zero coordinate error	nonzero coordinates incorrectly replaced with 0.0.	*z* = −0.232 → 0.0 (de Santis, 1987)^87^
count/index error	wrong extraction of discrete counts (sites, site types).	NSitetypes = 2 → 8 (Muñoz-Muñoz, 2017)^77^
orientation parameter error	misextraction of dipole and quadrupole orientation.	theta = 30.0 → 45.0 (Gao, 1997)^81^
coordinate extraction error	incorrect coordinates (nonzero to nonzero).	*z* = −0.232 → 1.185 (de Santis, 1987)^87^
missing parameter	parameter completely absent from extraction.	mass = 16.042 → (missing) (Koyama, 2005)^86^
electrostatic property misread	misreading of physical properties that describe electrostatic interactions (charges, dipoles, etc.).	dipole = 3.228 → 1.988 (Gao, 1997)^81^
categorical misclassification	site types or labels incorrectly classified.	SiteType = Dipole → LJ126 (Muñoz–Muñoz, 2017)^77^

a Examples are shown in the format *reference* → *extracted.*

**Table 5:** Distribution of Extraction Error Types across 4632 Total Errors from 21 Scientific Papers for All Force Fields and Replica Runs

**error type**	**count**	**%**	**num of papers affected**
missing parameter	2934	63.3%	13
numeric value mismatch	469	10.1%	13
count/index error	429	9.3%	16
coordinate extraction error	416	9.0%	9
zero coordinate error	220	4.7%	7
electrostatic property misread	116	2.5%	5
orientation parameter error	30	0.6%	1
categorical misclassification	18	0.4%	1
**total**	**4632**		

Furthermore, specific force field parameters that were most frequently extracted incorrectly are summarized in Table 6. Basic physical properties such as “mass” were the most common source of error (16.0%), often due to “Missing Parameter” or “Numeric Value Mismatch”. Other frequently erroneous variables included “NSiteTypes” (8.6%), “dipole” (6.6%), and “theta” (5.8%). Coordinates (“*z*”, “*y*”, “*x*”) also contributed to the error count, primarily susceptible to “Coordinate Extraction Error” or by missing entirely.

**Table 6:** Molecular Parameters Most Frequently Extracted Incorrectly, Showing the 15 Variables with the Highest Error Counts

**variable**	**error count**	**%**	**primary error types**
mass	740	16.0%	numeric value mismatch, missing parameter
*z*	680	14.7%	missing parameter, coordinate extraction error
*x*	640	13.8%	missing parameter, coordinate extraction error
NSiteTypes	399	8.6%	count/index error, missing parameter
*y*	387	8.4%	missing parameter, coordinate extraction error
NSites	340	7.3%	missing parameter, count/index error
dipole	304	6.6%	missing parameter, electrostatic property misread
SiteType	289	6.2%	missing parameter, categorical misclassification
theta	267	5.8%	missing parameter, orientation parameter error
phi	237	5.1%	missing parameter
sigma	145	3.1%	missing parameter, numeric value mismatch
epsilon	132	2.8%	missing parameter, numeric value mismatch
quadrupole	46	1.0%	electrostatic property misread, missing parameter
charge	22	0.5%	missing parameter
MIE_m	2	0.0%	missing parameter

## Conclusions

LLMs provide a remarkably fast and robust route for digitizing force field models from publications. The LLM-assisted data extraction pipeline proposed in this work transforms the model data contained in research paper .pdf files into structured, machine-readable input files. This automated approach offers a significant improvement over manual methods, enhancing efficiency and reducing the potential for human errors in preparing molecular simulation inputs. The extraction accuracy of the LLM-assisted method is roughly 77 to 94%—depending on the employed LLM. The overall best performance was achieved by the Gemini 2.5 Pro LLM with an extraction accuracy of 94.0%. Performance variability across papers and occasional outliers further highlight the need to study model stability and generalization in more detail.

Accurate and reproducible molecular simulations demand 100% accuracy, i.e., force field input files without any errors. Thus, the method and results from this paper should be regarded as a starting point for developing a reliable and autonomous digitization tool. The method proposed in this work is an attractive alternative to tedious and error-prone “by hand” digitization of force fields for molecular simulations.

A further limitation is that both the source publications and the ground-truth reference files are publicly available and may have been used as training data for the proprietary LLMs tested in this work, which could have influenced the reported extraction accuracy through memorization rather than genuine understanding and extraction of the provided input. Future studies and validation on newly published or unpublished force field data could help rule out any possible training data contamination.

Possible ways to improve the accuracy of the digitization method can be guided by the experiences from the comprehensive evaluation carried out in this work. These results provide a robust benchmark for testing refinements and improvements. A large variety of routes to improve the promising results from this work seem to be attractive. On the one hand, the prompt may be further refined to give more context in the translation between the Z-matrix and Cartesian coordinates or by providing more physical background/information. The error analysis revealed that numeric mismatches and missing parameters are the most frequent error types, which most frequently occur for mass and coordinates. On the other hand, OCR is not 100% error-free, and newer, even stronger LLMs might handle this task better. Additionally, LLMs with greater variability or higher error rates may benefit from ensembling, further prompt adaptation, or supplementary rule-based checks. Moreover, another interesting approach would be to use different LLMs sequentially.

Furthermore, the comprehensive evaluation of the new method provided insights into challenges in force field publication in the scientific literature due to a lack of standardization: Different unit systems are used in different publications (and communities); different coordinate systems are used (Z-matrix, Cartesian, etc.); information on a given force field is often scattered across a publication, with some data given in the bulk text, some in tables, and sometimes even in graphics. Sometimes, no spatial coordinates are specified but have to be determined as the equilibrium configuration from the stated intramolecular interactions. Moreover, there is no universal and standardized force field data format established yet. These insights highlight not only the need for more advanced extraction methods but also the need for community-wide standards and publication practices to make force fields consistently reusable.

## Supplementary Material





## Data Availability

The Python pipeline developed within this work is provided in the Supporting Information. Moreover, examples of force field .pm files built within this work using the LLMs are provided, as well as the reference .pm files that were built “by hand”.

## Notes

Complete Jupyter notebook (molecular_engineering_openrouter.ipynb) implementing the five-step digitization method, manually curated reference .pm files serving as ground-truth for the accuracy evaluation, and examples of generated .pm files produced by the LLM-based extraction pipeline (ZIP)
Definitions of the error types introduced in the main manuscript, extraction accuracy metrics excluding the publication contributing the majority of force fields, and information on the use of the Jupyter notebook (PDF)
