# Supplementary material for: Toward Autonomous Extraction and Building of Machine-Readable Molecular Models from Publications Using Large Language Models
Source: J Chem Inf Model. 2026 Oct 6;66(20):13383–96. doi: 10.1021/acs.jcim.6c02128 (PMC13715525; doi:10.1021/acs.jcim.6c02128)
Supplement: Supplementary file 1 [file ci6c02128_si_002.pdf]

Supporting Information

Toward Autonomous Extraction and Building of Machine-Readable Molecular Models from Publications Using Large Language Models

Florian Fleckenstein[a], *Volodymyr Rodin*[b], *Isabell Dieudonné*[a], *Fadi Al Machot*[c], *Silvia Chiacchiera*[d], *Martin Horsch*[c], *Amila Akagic*[b] *and Simon Stephan*[a,*]

[a]Laboratory of Heat- and Mass Transfer, Otto von Guericke University Magdeburg, 39106 Magdeburg, Germany

[b]Department of Computer Science and Informatics, University of Sarajevo, 71000 Sarajevo, Bosnia and Herzegovina

[c]Department of Data Science, Norwegian University of Life Sciences, 1432 Ås, Norway

[d]UKRI Science and Technology Facilities Council, Daresbury Laboratory, WA4 4AD Warrington, United Kingdom

*simon.stephan@ovgu.de

2026-09-29

Supporting Information Contents

The Supporting Information for the publication *Toward Autonomous Extraction and Building of Machine-Readable Molecular Models from Publications Using Large Language Models* contains the following components:

- Manually curated reference `.pm` files serving as ground truth for accuracy evaluation (given in the electronic Supporting Information),
- Examples of automatically generated `.pm` files produced by the LLM-based extraction pipeline (given in the electronic Supporting Information),
- Definitions of the error types introduced within the main manuscript,
- Extraction accuracy metrics excluding the publication contributing the majority of force fields in the main manuscript,
- Complete Jupyter notebook (`molecular_engineering_openrouter.ipynb`) implementing the five-step digitization method outlined in the main body of this work.

Definitions of Error Types

As outlined in the main manuscript, several different types of errors occurred in the LLM-assisted extraction procedure. These errors are classified in Table 4, and the relative share of each error type is shown in Table 5 in the main body of this work. In general, errors were distinguished between incorrectly identified parameters and missing parameters. The incorrectly identified parameters were further categorized as follows:

(1) Errors related to the coordinate system. These either involved extracted values that differed from the reference values ("Coordinate Extraction Error") or cases in which coordinates were replaced by the value zero ("Zero Coordinate Error").

(2) Errors related to the classification of properties within the `.pm` file format. A "Count/Index Error" was assigned when a property specific to the `.pm` file format (such as `NSiteTypes`) was given an incorrect value. "Categorical Misclassification" was used when the extracted value or string did not match the expected property type, for example, `Dipole` instead of `LJ126`.

(3) Errors associated with incorrectly extracted physical values. If the orientation of dipole or quadrupole moments was incorrect, the error was classified as an "Orientation Parameter Error". If the magnitude of electrostatic properties (`Charge`, `Dipole`, and `Quadrupole`) was incorrect, the error was labeled "Electrostatic Property Misread". Other incorrectly extracted properties were classified as "Numeric Value Mismatch".

In general, no further distinction was made between slightly and substantially deviating extracted values; however, most incorrect values differed by an order of magnitude or in sign.

Extraction Accuracy Metrics Excluding the Publication Contributing the Majority of Force Fields

Given that one publication (#21, cf. Table 2 of the main manuscript) accounts for 62 of the 114 tested force field models and approximately 40% of the total parameters, the sensitivity of the reported results to this publication's influence is reported in Table S1 excluding publication #21. The overall extraction accuracy remains close to the values reported above, with small, consistent shifts: The three best-performing models (Gemini 2.5 Pro, Claude 4 Sonnet, Claude 3.7 Sonnet) show slightly lower accuracy, while the two lowest-performing models (Gemini 2.5 Flash, GPT-4o) show slightly higher accuracy, when this publication is excluded. Nevertheless, the relative ranking of all five LLMs remains unchanged.

Table S1: Summary of performance metrics results for studied LLMs from the validation data set, excluding publication #21. The average accuracy was computed by calculating the mean accuracy over papers; correct/incorrect/missing counts are aggregate totals of parameters across all papers and runs.

**Model**	**Avg Acc / %**	***P* / %**	***R* / %**	***F*1 / %**	**Correct**	**Incorrect**	**Missing**
Gemini 2.5 Pro	**93.7**	**95.7**	**94.2**	**95.0**	4540 (94%)	202 (4%)	76 (2%)
Claude 4 Sonnet	91.9	93.8	93.4	93.6	4501 (93%)	299 (6%)	18 (0%)
Claude 3.7 Sonnet	91.7	94.2	93.7	94.0	4515 (94%)	276 (6%)	27 (1%)
Gemini 2.5 Flash	90.3	94.7	92.6	93.6	4463 (93%)	251 (5%)	104 (2%)
GPT-4o	77.7	93.5	78.6	85.4	3788 (79%)	265 (6%)	765 (16%)
Average	-	-	-	-	- (91%)	- (5%)	- (4%)

Running the Colab Notebook

The electronic Supporting Information includes a Jupyter notebook (`molecular_engineering_openrouter.ipynb`) that implements the complete digitization pipeline described below. To reproduce the results or process new publications, follow these configuration steps:

API Key Configuration

The notebook requires two API keys for the external services used in the pipeline:

Mistral OCR API Key

In Section 2 (Cell 4), replace the placeholder API key in the following line:

```
client = Mistral(api_key="YOUR_MISTRAL_API_KEY_HERE")
```

OpenRouter API Key

In Section 7 (Cell with "Run Parameter Extraction"), replace the placeholder API key:

```
api_key = "YOUR_OPENROUTER_API_KEY_HERE"
```

Directory Configuration

Set the `extract_dir` variable to point to your directory containing PDF files:

For OCR Processing (Section 2)

Modify the directory path in Cell 5:

```
extract_dir = "path/to/your/papers"
```

For Parameter Extraction (Section 7)

Update the extraction directory in the same section:

```
extract_dir = "path/to/your/papers"
```

For Evaluation and Visualization (Sections 9 and 11)

Ensure the directory path is consistent in both evaluation sections:

```
extract_dir = "path/to/your/papers"
```

Model Selection and File Pattern Configuration

Model Selection (Section 7)

Specify the desired LLM for parameter extraction by modifying the `selected_model` variable:

```
selected_model = "anthropic/claude-3.7-sonnet"
selected_model = "google/gemini-2.5-pro"
selected_model = "openai/gpt-4o"
```

Evaluation File Pattern (Section 8)

After running Section 7, update the file pattern in Section 8 to match the generated extraction files. Modify line 23 in the `find_files_to_compare` function:

```
extraction_files = glob.glob(os.path.join(subfolder_path,
    "*_openrouter_[model_name]_[version].txt"))
```

Replace `[model_name]` and `[version]` with the actual values from your extraction run (e.g., `*_openrouter_anthropic_claude-sonnet-4_3.txt`).

PM File Generation Pattern (Section 6)

For generating `.pm` files from extraction results, update the model pattern in the `generate_pm_files_from_extractions` function call:

```
generated_files = generate_pm_files_from_extractions(
    extract_dir,
    model_pattern="*_openrouter_[model_name]_[version].txt"
)
```

Execution Order

Execute the notebook sections sequentially:

1. Section 1: Upload and extract PDF files
2. Section 2: OCR processing with Mistral AI
3. Sections 3-6: Text cleaning and table extraction
4. Section 7: Parameter extraction with selected LLM
5. Section 8: Configure evaluation file patterns based on Section 7 output
6. Section 9: Accuracy evaluation against reference files
7. Sections 10-11: Visualization of results

The notebook automatically handles file naming conventions, ensuring that extraction results are properly matched with reference files during evaluation. Output files follow the naming pattern `[paper]_openrouter_[model]_[version].txt`, enabling systematic comparison across different models and multiple runs. The model name and version number in the filename must be updated in the evaluation and PM generation sections to match the files produced by the parameter extraction step.
